# Bis(3-benzoyl-1,1-di-*sec*-butyl­thio­ureato-κ^2^ *O*,*S*)palladium(II)

**DOI:** 10.1107/S1600536811036853

**Published:** 2011-09-17

**Authors:** N. Selvakumaran, R. Karvembu, Seik Weng Ng, Edward R. T. Tiekink

**Affiliations:** a Department of Chemistry, National Institute of Technology, Tiruchirappalli 620 015, India; b Department of Chemistry, University of Malaya, 50603 Kuala Lumpur, Malaysia; c Chemistry Department, Faculty of, Science, King Abdulaziz University, PO Box 80203 Jeddah, Saudi Arabia

## Abstract

The complex mol­ecule of the title complex, [Pd(C_16_H_23_N_2_OS)_2_], is completed by crystallographic twofold symmetry with the metal atom lying on the rotation axis. The Pd^II^ atom exists within a slightly distorted square-planar geometry defined by a *cis*-O_2_S_2_ donor set. The dihedral angle formed between the mean planes of the symmetry-related six-membered chelate rings is 12.88 (7)° and the bond lengths within the rings are indicative of significant electron delocalization. In the crystal, mol­ecules aggregate into dimers linked by four C—H⋯O inter­actions.

## Related literature

For background to the synthesis and cytotoxicity of related Pd^II^ complexes of *N*,*N*-di(alk­yl/ar­yl)-*N*′-benzoyl­thio­urea ligands, see: Selvakumaran *et al.* (2011 ▶). 
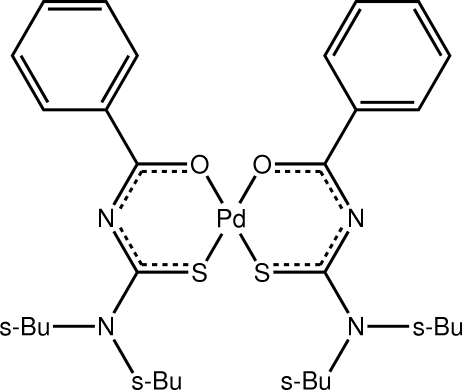


## Experimental

### 

#### Crystal data

[Pd(C_16_H_23_N_2_OS)_2_]
*M* *_r_* = 689.27
Tetragonal, 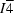

*a* = 13.2737 (1) Å
*c* = 19.5597 (5) Å
*V* = 3446.25 (9) Å^3^
*Z* = 4
Mo *K*α radiation
μ = 0.69 mm^−1^
*T* = 100 K
0.30 × 0.25 × 0.20 mm

#### Data collection

Agilent SuperNova Dual diffractometer with an Atlas detector
Absorption correction: multi-scan (*CrysAlis PRO*; Agilent, 2010 ▶) *T*_min_ = 0.819, *T*_max_ = 0.874
4752 measured reflections
3229 independent reflections
3152 reflections with *I* > 2σ(*I*)
*R*_int_ = 0.020

#### Refinement

*R*[*F* ^2^ > 2σ(*F* ^2^)] = 0.023
*wR*(*F* ^2^) = 0.058
*S* = 1.02
3229 reflections
186 parameters
H-atom parameters constrained
Δρ_max_ = 0.51 e Å^−3^
Δρ_min_ = −0.44 e Å^−3^
Absolute structure: Flack (1983 ▶), 1225 Friedel pairs
Flack parameter: −0.02 (2)

### 

Data collection: *CrysAlis PRO* (Agilent, 2010 ▶); cell refinement: *CrysAlis PRO*; data reduction: *CrysAlis PRO*; program(s) used to solve structure: *SHELXS97* (Sheldrick, 2008 ▶); program(s) used to refine structure: *SHELXL97* (Sheldrick, 2008 ▶); molecular graphics: *ORTEP-3* (Farrugia, 1997 ▶) and *DIAMOND* (Brandenburg, 2006 ▶); software used to prepare material for publication: *publCIF* (Westrip, 2010 ▶).

## Supplementary Material

Crystal structure: contains datablock(s) global, I. DOI: 10.1107/S1600536811036853/hb6404sup1.cif

Structure factors: contains datablock(s) I. DOI: 10.1107/S1600536811036853/hb6404Isup2.hkl

Additional supplementary materials:  crystallographic information; 3D view; checkCIF report

## Figures and Tables

**Table:** 

Pd—O1	2.0230 (17)
Pd—S1	2.2497 (6)

**Table:** 

O1—Pd—S1	93.76 (5)

**Table 2:** Hydrogen-bond geometry (Å, °)

*D*—H⋯*A*	*D*—H	H⋯*A*	*D*⋯*A*	*D*—H⋯*A*
C6—H6⋯O1^i^	0.95	2.43	3.179 (3)	136

Symmetry code: (i) 
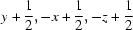
.

**Fig. 1.**
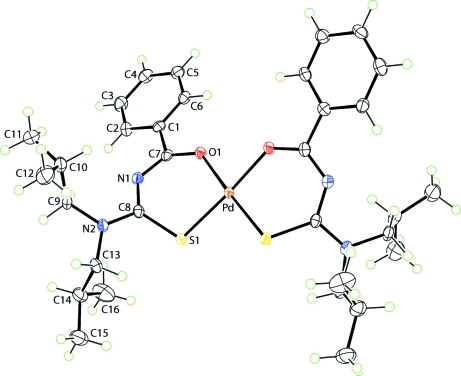
The molecular structure of (I) showing displacement ellipsoids at the 50% probability level. The molecule has 2-fold symmetry. Unlabelled atoms are related by the symmetry operation 1 - x, -y, z.

**Fig. 2.**
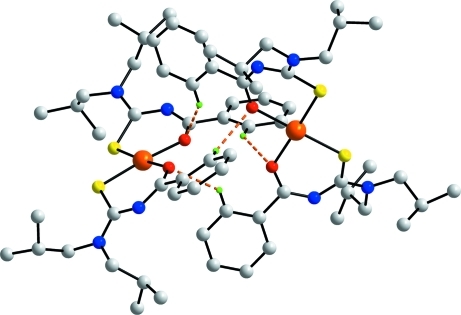
Two molecule aggregate in (I) mediated by C—H···O interactions shown as orange dashed lines. Hydrogen atoms not participating in C—H···O contacts have been omitted for reasons of clarity.

## supplementary crystallographic information

### Comment

The title complex, (I), was investigated during a study of the synthesis and cytotoxicity profiles of *N*,*N*-di(alkyl/aryl)-*N*'-benzoylthiourea ligands, LH (Selvakumaran *et al.*, 2011).

The Pd^II^ atom in (I), Fig. 1, exists in a square planar geometry defined by a *cis*-O_2_S_2_ donor set, Table 1, as found for related Pd*L*_2_ species (Selvakumaran *et al.*, 2011). The molecule has crystallographically imposed 2-fold symmetry. There are significant deviations from the least-squares plane through the six-membered chelate ring (r.m.s. deviation = 0.233 Å) with the maximum deviations being found for the S1 (0.255 (1) Å) and Pd (–0.163 (1) Å) atoms. The major twist in the ring is found about the N1—C8 bond as seen in the value of the C7—N1—C8—S1 torsion angle of -19.3 (4) °. Nevertheless, the bond distance data, Table 1, are consistent with considerable delocalization of π-electron density over the six atoms; a similar conclusion was made for related species (Selvakumaran *et al.*, 2011). The dihedral angle formed between the symmetry related chelate rings is 12.88 (7) °.

The most prominent intermolecular interactions in the crystal structure are of the type C—H···O, Table 2. These involve benzene-H and the coordinated O atoms, and result in the formation of two molecule aggregates sustained, from symmetry, by four such interactions, Fig. 2.

### Experimental

The title complex, (I), was prepared and characterized as in the literature (Selvakumaran *et al.*, 2011). Orange blocks were obtained  by slow evaporation of  a dichloromethane solution of the complex.

### Refinement

The H-atoms were placed in calculated positions (C—H 0.95 to 1.00 Å) and were included in the refinement in the riding model approximation, with *U*_iso_(H) set to 1.2 to 1.5*U*_equiv_(C).

### Crystal data

**Table:**

[Pd(C_16_H_23_N_2_OS)_2_]	*D*_x_ = 1.328 Mg m^−^^3^
*M**_r_* = 689.27	Mo *K*α radiation, λ = 0.71073 Å
Tetragonal, *I*4	Cell parameters from 3599 reflections
Hall symbol: I -4	θ = 3.0–29.3°
*a* = 13.2737 (1) Å	µ = 0.69 mm^−^^1^
*c* = 19.5597 (5) Å	*T* = 100 K
*V* = 3446.25 (9) Å^3^	Block, orange
*Z* = 4	0.30 × 0.25 × 0.20 mm
*F*(000) = 1440	

### Data collection

**Table:**

Agilent SuperNova Dual diffractometer with an Atlas detector	3229 independent reflections
Radiation source: SuperNova (Mo) X-ray Source	3152 reflections with *I* > 2σ(*I*)
Mirror	*R*_int_ = 0.020
Detector resolution: 10.4041 pixels mm^-1^	θ_max_ = 27.5°, θ_min_ = 3.0°
ω scans	*h* = −17→10
Absorption correction: multi-scan (*CrysAlis PRO*; Agilent, 2010)	*k* = −14→13
*T*_min_ = 0.819, *T*_max_ = 0.874	*l* = −16→25
4752 measured reflections	

### Refinement

**Table:**

Refinement on *F*^2^	Secondary atom site location: difference Fourier map
Least-squares matrix: full	Hydrogen site location: inferred from neighbouring sites
*R*[*F*^2^ > 2σ(*F*^2^)] = 0.023	H-atom parameters constrained
*wR*(*F*^2^) = 0.058	*w* = 1/[σ^2^(*F*_o_^2^) + (0.0272*P*)^2^ + 1.3893*P*] where *P* = (*F*_o_^2^ + 2*F*_c_^2^)/3
*S* = 1.02	(Δ/σ)_max_ < 0.001
3229 reflections	Δρ_max_ = 0.51 e Å^−^^3^
186 parameters	Δρ_min_ = −0.44 e Å^−^^3^
0 restraints	Absolute structure: Flack (1983), 1225 Friedel pairs
Primary atom site location: structure-invariant direct methods	Flack parameter: −0.02 (2)

### Special details

**Table:**

Geometry. All e.s.d.'s (except the e.s.d. in the dihedral angle between two l.s. planes) are estimated using the full covariance matrix. The cell e.s.d.'s are taken into account individually in the estimation of e.s.d.'s in distances, angles and torsion angles; correlations between e.s.d.'s in cell parameters are only used when they are defined by crystal symmetry. An approximate (isotropic) treatment of cell e.s.d.'s is used for estimating e.s.d.'s involving l.s. planes.
Refinement. Refinement of *F*^2^ against ALL reflections. The weighted *R*-factor *wR* and goodness of fit *S* are based on *F*^2^, conventional *R*-factors *R* are based on *F*, with *F* set to zero for negative *F*^2^. The threshold expression of *F*^2^ > σ(*F*^2^) is used only for calculating *R*-factors(gt) *etc*. and is not relevant to the choice of reflections for refinement. *R*-factors based on *F*^2^ are statistically about twice as large as those based on *F*, and *R*- factors based on ALL data will be even larger.

### Fractional atomic coordinates and isotropic or equivalent isotropic displacement parameters (Å^2^)

**Table:**

	*x*	*y*	*z*	*U*_iso_*/*U*_eq_	
Pd	0.5000	0.0000	0.076929 (11)	0.01307 (7)	
S1	0.60853 (5)	0.04633 (5)	−0.00563 (3)	0.01761 (13)	
O1	0.59796 (13)	0.03016 (13)	0.15347 (9)	0.0178 (4)	
N1	0.72361 (18)	0.12436 (17)	0.09978 (10)	0.0178 (5)	
N2	0.72955 (17)	0.20604 (16)	−0.00131 (11)	0.0183 (5)	
C1	0.7459 (2)	0.06393 (19)	0.21341 (13)	0.0176 (5)	
C2	0.8469 (2)	0.0954 (2)	0.21380 (14)	0.0215 (6)	
H2	0.8761	0.1232	0.1737	0.026*	
C3	0.9035 (2)	0.0857 (2)	0.27244 (15)	0.0270 (6)	
H3	0.9721	0.1060	0.2723	0.032*	
C4	0.8614 (2)	0.0468 (2)	0.33146 (14)	0.0248 (6)	
H4	0.9009	0.0404	0.3717	0.030*	
C5	0.7609 (2)	0.0169 (2)	0.33165 (13)	0.0231 (6)	
H5	0.7315	−0.0094	0.3722	0.028*	
C6	0.7041 (2)	0.02554 (19)	0.27269 (13)	0.0193 (5)	
H6	0.6356	0.0049	0.2729	0.023*	
C7	0.68364 (19)	0.07145 (19)	0.15006 (13)	0.0164 (5)	
C8	0.68990 (19)	0.13061 (19)	0.03510 (13)	0.0162 (5)	
C9	0.7978 (2)	0.2781 (2)	0.03246 (15)	0.0223 (6)	
H9A	0.8344	0.3166	−0.0030	0.027*	
H9B	0.8482	0.2400	0.0593	0.027*	
C10	0.7437 (2)	0.3523 (2)	0.07997 (17)	0.0274 (6)	
H10	0.6951	0.3133	0.1087	0.033*	
C11	0.8185 (3)	0.4033 (3)	0.12724 (16)	0.0361 (8)	
H11A	0.8555	0.3521	0.1531	0.054*	
H11B	0.7824	0.4475	0.1590	0.054*	
H11C	0.8660	0.4434	0.1001	0.054*	
C12	0.6836 (3)	0.4300 (3)	0.03869 (18)	0.0389 (8)	
H12A	0.6494	0.4763	0.0700	0.058*	
H12B	0.6334	0.3952	0.0105	0.058*	
H12C	0.7295	0.4680	0.0091	0.058*	
C13	0.71911 (19)	0.21523 (19)	−0.07512 (15)	0.0198 (5)	
H13A	0.7031	0.2861	−0.0865	0.024*	
H13B	0.6618	0.1731	−0.0903	0.024*	
C14	0.8141 (2)	0.1834 (2)	−0.11491 (15)	0.0275 (6)	
H14	0.8674	0.2352	−0.1065	0.033*	
C15	0.7901 (3)	0.1834 (3)	−0.19190 (14)	0.0362 (8)	
H15A	0.8503	0.1637	−0.2176	0.054*	
H15B	0.7690	0.2510	−0.2059	0.054*	
H15C	0.7356	0.1354	−0.2011	0.054*	
C16	0.8549 (3)	0.0821 (3)	−0.09144 (17)	0.0445 (10)	
H16A	0.8704	0.0851	−0.0425	0.067*	
H16B	0.9164	0.0662	−0.1170	0.067*	
H16C	0.8043	0.0297	−0.0997	0.067*	

### Atomic displacement parameters (Å^2^)

**Table:**

	*U*^11^	*U*^22^	*U*^33^	*U*^12^	*U*^13^	*U*^23^
Pd	0.01199 (15)	0.01268 (15)	0.01453 (11)	−0.00124 (12)	0.000	0.000
S1	0.0181 (3)	0.0180 (3)	0.0167 (3)	−0.0044 (2)	0.0020 (3)	−0.0003 (3)
O1	0.0138 (9)	0.0227 (10)	0.0169 (8)	−0.0038 (8)	−0.0002 (8)	0.0001 (8)
N1	0.0168 (11)	0.0171 (11)	0.0196 (11)	−0.0019 (9)	0.0001 (8)	−0.0007 (8)
N2	0.0183 (11)	0.0154 (11)	0.0213 (11)	−0.0027 (8)	0.0033 (10)	0.0004 (10)
C1	0.0185 (13)	0.0148 (13)	0.0196 (13)	0.0031 (10)	−0.0001 (11)	−0.0035 (10)
C2	0.0191 (14)	0.0200 (14)	0.0254 (15)	−0.0028 (11)	0.0005 (12)	0.0022 (12)
C3	0.0167 (13)	0.0314 (15)	0.0328 (15)	−0.0021 (12)	−0.0033 (12)	−0.0026 (12)
C4	0.0231 (15)	0.0270 (16)	0.0242 (15)	0.0021 (12)	−0.0096 (12)	−0.0014 (12)
C5	0.0254 (15)	0.0221 (15)	0.0220 (14)	−0.0004 (12)	−0.0016 (11)	−0.0012 (11)
C6	0.0155 (13)	0.0198 (13)	0.0226 (12)	−0.0005 (10)	−0.0028 (10)	−0.0019 (10)
C7	0.0169 (13)	0.0123 (12)	0.0201 (12)	0.0013 (10)	0.0014 (11)	−0.0044 (11)
C8	0.0120 (12)	0.0154 (13)	0.0212 (12)	0.0004 (10)	0.0021 (10)	0.0001 (11)
C9	0.0240 (15)	0.0176 (14)	0.0255 (14)	−0.0096 (11)	0.0050 (12)	−0.0020 (12)
C10	0.0306 (15)	0.0201 (14)	0.0315 (14)	−0.0064 (11)	0.0099 (15)	−0.0023 (14)
C11	0.047 (2)	0.0327 (18)	0.0289 (16)	−0.0097 (15)	0.0074 (15)	−0.0085 (14)
C12	0.044 (2)	0.0269 (17)	0.0458 (19)	0.0035 (15)	0.0043 (17)	−0.0046 (15)
C13	0.0205 (13)	0.0175 (13)	0.0214 (12)	−0.0003 (10)	0.0013 (13)	0.0050 (13)
C14	0.0270 (16)	0.0302 (17)	0.0252 (14)	−0.0007 (13)	0.0067 (13)	0.0000 (13)
C15	0.045 (2)	0.0394 (19)	0.0241 (15)	−0.0005 (16)	0.0056 (14)	0.0023 (14)
C16	0.045 (2)	0.046 (2)	0.042 (2)	0.0209 (17)	0.0167 (16)	0.0055 (16)

### Geometric parameters (Å, °)

**Table:**

Pd—O1^i^	2.0230 (17)	C9—H9A	0.9900
Pd—O1	2.0230 (17)	C9—H9B	0.9900
Pd—S1^i^	2.2497 (6)	C10—C11	1.517 (4)
Pd—S1	2.2497 (6)	C10—C12	1.533 (5)
S1—C8	1.747 (3)	C10—H10	1.0000
O1—C7	1.264 (3)	C11—H11A	0.9800
N1—C7	1.320 (3)	C11—H11B	0.9800
N1—C8	1.344 (3)	C11—H11C	0.9800
N2—C8	1.337 (3)	C12—H12A	0.9800
N2—C13	1.455 (3)	C12—H12B	0.9800
N2—C9	1.473 (3)	C12—H12C	0.9800
C1—C6	1.383 (3)	C13—C14	1.541 (4)
C1—C2	1.404 (4)	C13—H13A	0.9900
C1—C7	1.493 (3)	C13—H13B	0.9900
C2—C3	1.377 (4)	C14—C16	1.520 (5)
C2—H2	0.9500	C14—C15	1.539 (4)
C3—C4	1.383 (4)	C14—H14	1.0000
C3—H3	0.9500	C15—H15A	0.9800
C4—C5	1.391 (4)	C15—H15B	0.9800
C4—H4	0.9500	C15—H15C	0.9800
C5—C6	1.383 (4)	C16—H16A	0.9800
C5—H5	0.9500	C16—H16B	0.9800
C6—H6	0.9500	C16—H16C	0.9800
C9—C10	1.533 (4)		
			
O1^i^—Pd—O1	84.53 (10)	C11—C10—C9	110.5 (3)
O1^i^—Pd—S1^i^	93.76 (5)	C11—C10—C12	111.2 (2)
O1—Pd—S1^i^	175.49 (5)	C9—C10—C12	110.9 (3)
O1^i^—Pd—S1	175.49 (5)	C11—C10—H10	108.1
O1—Pd—S1	93.76 (5)	C9—C10—H10	108.1
S1^i^—Pd—S1	88.26 (3)	C12—C10—H10	108.1
C8—S1—Pd	104.10 (9)	C10—C11—H11A	109.5
C7—O1—Pd	128.68 (16)	C10—C11—H11B	109.5
C7—N1—C8	126.9 (2)	H11A—C11—H11B	109.5
C8—N2—C13	123.6 (2)	C10—C11—H11C	109.5
C8—N2—C9	119.3 (2)	H11A—C11—H11C	109.5
C13—N2—C9	116.7 (2)	H11B—C11—H11C	109.5
C6—C1—C2	119.2 (2)	C10—C12—H12A	109.5
C6—C1—C7	119.9 (2)	C10—C12—H12B	109.5
C2—C1—C7	120.9 (2)	H12A—C12—H12B	109.5
C3—C2—C1	119.9 (3)	C10—C12—H12C	109.5
C3—C2—H2	120.1	H12A—C12—H12C	109.5
C1—C2—H2	120.1	H12B—C12—H12C	109.5
C2—C3—C4	120.6 (3)	N2—C13—C14	113.6 (2)
C2—C3—H3	119.7	N2—C13—H13A	108.8
C4—C3—H3	119.7	C14—C13—H13A	108.8
C3—C4—C5	119.8 (3)	N2—C13—H13B	108.8
C3—C4—H4	120.1	C14—C13—H13B	108.8
C5—C4—H4	120.1	H13A—C13—H13B	107.7
C6—C5—C4	119.8 (3)	C16—C14—C15	111.7 (3)
C6—C5—H5	120.1	C16—C14—C13	112.4 (2)
C4—C5—H5	120.1	C15—C14—C13	108.9 (3)
C1—C6—C5	120.7 (2)	C16—C14—H14	107.9
C1—C6—H6	119.6	C15—C14—H14	107.9
C5—C6—H6	119.6	C13—C14—H14	107.9
O1—C7—N1	129.2 (2)	C14—C15—H15A	109.5
O1—C7—C1	115.2 (2)	C14—C15—H15B	109.5
N1—C7—C1	115.6 (2)	H15A—C15—H15B	109.5
N2—C8—N1	114.6 (2)	C14—C15—H15C	109.5
N2—C8—S1	118.7 (2)	H15A—C15—H15C	109.5
N1—C8—S1	126.5 (2)	H15B—C15—H15C	109.5
N2—C9—C10	113.6 (2)	C14—C16—H16A	109.5
N2—C9—H9A	108.8	C14—C16—H16B	109.5
C10—C9—H9A	108.8	H16A—C16—H16B	109.5
N2—C9—H9B	108.8	C14—C16—H16C	109.5
C10—C9—H9B	108.8	H16A—C16—H16C	109.5
H9A—C9—H9B	107.7	H16B—C16—H16C	109.5
			
O1^i^—Pd—S1—C8	42.5 (7)	C2—C1—C7—O1	172.0 (2)
O1—Pd—S1—C8	−24.95 (10)	C6—C1—C7—N1	168.8 (2)
S1^i^—Pd—S1—C8	159.09 (10)	C2—C1—C7—N1	−11.2 (4)
O1^i^—Pd—O1—C7	−168.9 (2)	C13—N2—C8—N1	167.7 (2)
S1^i^—Pd—O1—C7	123.2 (6)	C9—N2—C8—N1	−4.7 (3)
S1—Pd—O1—C7	6.9 (2)	C13—N2—C8—S1	−8.0 (3)
C6—C1—C2—C3	1.4 (4)	C9—N2—C8—S1	179.60 (19)
C7—C1—C2—C3	−178.6 (2)	C7—N1—C8—N2	165.5 (2)
C1—C2—C3—C4	−1.0 (4)	C7—N1—C8—S1	−19.3 (4)
C2—C3—C4—C5	0.1 (4)	Pd—S1—C8—N2	−148.11 (19)
C3—C4—C5—C6	0.6 (4)	Pd—S1—C8—N1	36.8 (2)
C2—C1—C6—C5	−0.7 (4)	C8—N2—C9—C10	−73.8 (3)
C7—C1—C6—C5	179.2 (2)	C13—N2—C9—C10	113.2 (3)
C4—C5—C6—C1	−0.2 (4)	N2—C9—C10—C11	164.1 (3)
Pd—O1—C7—N1	15.7 (4)	N2—C9—C10—C12	−72.2 (3)
Pd—O1—C7—C1	−168.02 (16)	C8—N2—C13—C14	−102.4 (3)
C8—N1—C7—O1	−13.8 (4)	C9—N2—C13—C14	70.2 (3)
C8—N1—C7—C1	169.9 (2)	N2—C13—C14—C16	48.6 (3)
C6—C1—C7—O1	−8.0 (3)	N2—C13—C14—C15	172.9 (2)

Symmetry codes: (i) −*x*+1, −*y*, *z*.

### Hydrogen-bond geometry (Å, °)

**Table:**

*D*—H···*A*	*D*—H	H···*A*	*D*···*A*	*D*—H···*A*
C6—H6···O1^ii^	0.95	2.43	3.179 (3)	136

Symmetry codes: (ii) *y*+1/2, −*x*+1/2, −*z*+1/2.
